# Common cellular events occur during wound healing and organ regeneration in the sea cucumber *Holothuria glaberrima*

**DOI:** 10.1186/1471-213X-7-115

**Published:** 2007-10-18

**Authors:** José E San Miguel-Ruiz, José E García-Arrarás

**Affiliations:** 1 Department of Biology, University of Puerto Rico, Rio Piedras, Puerto Rico

## Abstract

**Background:**

All animals possess some type of tissue repair mechanism. In some species, the capacity to repair tissues is limited to the healing of wounds. Other species, such as echinoderms, posses a striking repair capability that can include the replacement of entire organs. It has been reported that some mechanisms, namely extracellular matrix remodeling, appear to occur in most repair processes. However, it remains unclear to what extent the process of organ regeneration, particularly in animals where loss and regeneration of complex structures is a programmed natural event, is similar to wound healing. We have now used the sea cucumber *Holothuria glaberrima *to address this question.

**Results:**

Animals were lesioned by making a 3–5 mm transverse incision between one of the longitudinal muscle pairs along the bodywall. Lesioned tissues included muscle, nerve, water canal and dermis. Animals were allowed to heal for up to four weeks (2, 6, 12, 20, and 28 days post-injury) before sacrificed. Tissues were sectioned in a cryostat and changes in cellular and tissue elements during repair were evaluated using classical dyes, immmuohistochemistry and phalloidin labeling. In addition, the temporal and spatial distribution of cell proliferation in the animals was assayed using BrdU incorporation. We found that cellular events associated with wound healing in *H. glaberrima *correspond to those previously shown to occur during intestinal regeneration. These include: (1) an increase in the number of spherule-containing cells, (2) remodeling of the extracellular matrix, (3) formation of spindle-like structures that signal dedifferentiation of muscle cells in the area flanking the lesion site and (4) intense cellular division occurring mainly in the coelomic epithelium after the first week of regeneration.

**Conclusion:**

Our data indicate that *H. glaberrima *employs analogous cellular mechanisms during wound healing and organ regeneration. Thus, it is possible that regenerative limitations in some organisms are due either to the absence of particular mechanisms associated with repair or the inability of activating the repair process in some tissues or stages.

## Background

For years, researchers have been perplexed by the differences in regeneration capabilities between animal species [1-6]. On the one hand, some species such as Planaria, Hydra or starfish can readily regenerate lost organs or body parts in a remarkable way. In contrast, other species, such as most vertebrates, Drosophila or *C. elegans *have limited regenerative capacities. Nonetheless, all species have some capacity to heal wounds produced by external factors encountered during their existence. What, if any, is the relationship between these two processes? There are some indications that these processes might differ at the cellular and molecular levels. For example, in sea urchins it has been suggested that healing of broken spines occurs by a morphallactic mechanism involving recruitment of differentiated cells, while regeneration of removed spines and pedicellaria occurs by an epimorphic process involving undifferentiated precursors [7]. And in zebra fish, studies on fin regeneration have found that a fish mutant (*dob*- devoid of blastema) fails to regenerate the fin but has normal wound healing responses [8]. More recently it has been shown that both wound healing and regeneration in the axolotl are dependent on epithelial/mesenchymal interactions, the formation of the wound epidermis, the restructuring of the extracellular matrix and other cellular/molecular events [9]. These similarities led these authors to suggest that the regeneration capacities of these amphibians is basically a process of superhealing[9].

A related question that surfaces is whether regenerative processes in animals where regeneration is a common occurrence that follows autotomy or programmed organ loss, differ from those associated with wound healing? This has been suggested to occur in some animals where autotomy usually occurs in localized breakage zones giving rise to the possibility that the regeneration capacities of cells within these zones differ from those of cells in other parts of the animal. In fact, it has been proposed that animals that autotomize organs or appendages, such as echinoderms and arthropods, show a higher potency for regeneration [10,11].

In answering these questions, it is crucial to determine what mechanisms are shared or differ between the processes of organ regeneration and that of wound healing. We have now used the holothurian or sea cucumber, *Holothuria glaberrima*, to study the relationship between the two processes. Holothurians are echinoderms, a group that comprise animals renowned for their regeneration capabilities [12,13]. When exposed to noxious stimuli, *H. glaberrima *undergoes a programmed evisceration process where most of the digestive tract and associated organs are expelled from the body cavity [14,15]. Following evisceration, it then regenerates new viscera, being the digestive system the first to be formed. Our group has used this model system extensively, showing that, following evisceration, a new intestine forms from a thickening in the remaining mesentery [14,15]. The initial intestinal primordium consists of a solid tube that connects the esophagus with the cloaca. Eventually, a lumen is formed and the tube is organized into a functional intestine. The process of intestinal regeneration in *H. glaberrima *includes, cell division, cell migration, extracellular matrix remodeling and muscle de-differentiation [14,16-19].

We now extend the use of this model system to study wound healing and to compare the events that take place during wound healing with those previously shown to occur during organ regeneration. To this end, a technique was developed to be able to cause an incision in the bodywall. In particular, the incision was made from the coelomic cavity side of the bodywall; a region that would normally not be subjected to wounds generated from the external environment and therefore cannot be considered a normal programmed event. The incision damaged nerve, muscle, dermal tissues and coelomic epithelia among others. Healing of the wound was then studied during the following 4 weeks. Changes in cellular and tissue elements during healing were evaluated using classical dyes, immmuohistochemistry and phalloidin labeling. Our results show that wound healing in these organisms makes use of the same mechanisms previously shown to occur during organ regeneration, to restore epithelial, dermal, and muscular tissues.

## Results

### Overview of bodywall regeneration – Macroscopic

Upon dissecting the animals, the wound was observed under the dissection microscope prior to removing the bodywall section for further processing. The wound by 2 dpi was easily recognized as an oval-shaped fissure, perpendicular to the long axis of the pair of longitudinal muscles, whose borders presented clean cut edges. Inside this gap some tattoo ink could be seen scattered in the tissues; as well, the coelomic fluid contained round bodies 2–4 mm in diameter full of tattoo ink. Some scar tissue was already visible at the injury site by 6 dpi, especially in the dermis of the wall; furthermore, the muscle terminals had a rather smooth appearance and were slanted toward their respective wound edges. At 12 dpi, a connection of provisional matrix that linked the two separated longitudinal muscle terminals was visible; this connection was particularly evident in the medial region of the longitudinal axis of the muscles. The amount of this provisional matrix at the injury site continued increasing and expanding laterally throughout the subsequent healing stages, serving eventually to seal the wound. By 20 dpi the cavity had almost been filled and now only two small oval-shaped apertures parallel to the longitudinal muscles were visible, one at each side of the medial plane of the longitudinal muscle pair. However, the composition and appearance of this matrix was not that of the surrounding muscular tissue, being more translucent and not as dense. The wound was completely sealed by 28 dpi; nonetheless, it could be identified after careful inspection of the tissue, being somewhat recessed in relation to the rest of the muscle. Aside from this, the muscles seemed completely healed and even had the characteristic furrow between them, below which runs the radial nerve and the water canal.

### Overview of bodywall regeneration – Microscopic

#### A. Bodywall staining with Toluidine Blue

**Figure 1:**
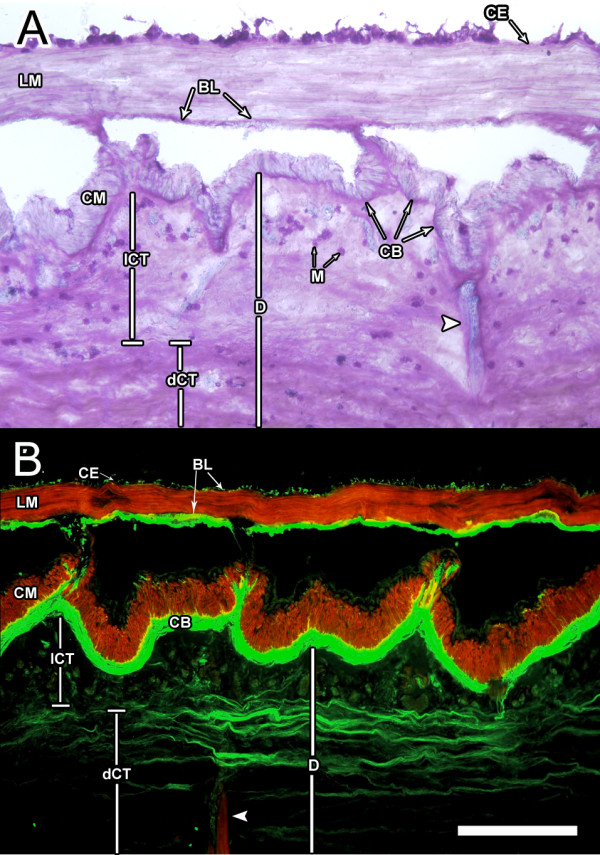
**Bodywall tissue organization**. Longitudinal sections of *H. glaberrima *bodywall depicting its major components, (A) stained with Toluidine blue and (B) double labeled with rhodamine-labeled phalloidin (red) and monoclonal antibody against holothurian collagen (HgCol, green). The main muscle systems and their relationship to the dense and loose connective tissue of the bodywall can be seen. BL-basal lamina of the coelomic epithelia, CB-collagen band, CE-coelomic epithelia, CM-circular muscle, D-dermis, including the loose (lCT) and dense (dCT) connective tissue, LM-longitudinal muscle, M-morulas. Arrowheads identify the ampulla within the bodywall. Bar = 300 μm.

In order to understand the changes that occur during wound healing, it is necessary to have a clear picture of the normal, non-injured bodywall. Fig. 1 provides two different, but complementary portrayals of the bodywall and its different tissues. Staining with the metachromatic stain Toluidine Blue the bodywall dermis, circular and longitudinal muscles and coelomic epithelium can be clearly identified. Alternatively, the use of other markers with fluorescent probes serves to specifically highlight particular tissues. In this case phalloidin labeling shows the muscle tissues while HgCol labeling shows the dense layer of collagen in the bodywall adjacent to the circular muscle.

**Figure 2:**
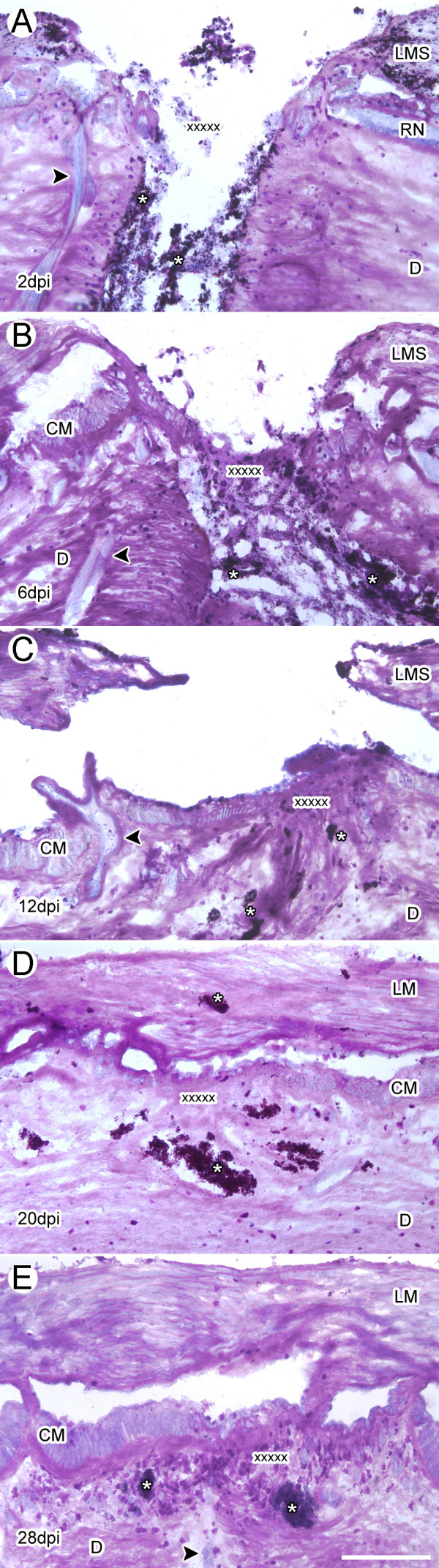
**Stages in wound healing following transection of *H. glaberrima *bodywall**. Longitudinal tissue sections of injured body wall at (A) 2, (B) 6, (C) 12, (D) 20 and (E) 28 days post injury (dpi) were stained with Toluidene Blue. (A) At 2 dpi the area is filled with debris and a clot has been formed. (B) By 6 dpi, the wound area is filled with a transitory matrix that appears to contain some of the clot material as well as new extracellular material. The muscle has healed and the stumps appear to be regenerating. (C) By 12 dpi the bodywall connective tissue has recovered some of its original organization, although its composition appears different from the non-injured tissue. The longitudinal muscle stumps have grown but have not rejoined. (D) By 20 dpi the connective tissue still appears slightly disorganized but the muscle stumps have connected forming a continuous longitudinal muscle. (E) At 28 dpi, both the muscle and the connective tissue have recovered much of the structure and organization found within non-injured tissue. CM-circular muscle, D-dermis, LM-longitudinal muscle, LMS-longitudinal muscle stump, RN-radial nerve. X's denote the injury site; Asterisks show the presence of tattoo ink used to label the injury site; arrowheads signal the ampulla structures within the bodywall. Bar = 300 μm.

Using Toluidine Blue, we were able to analyze the changes that occurred in the bodywall tissues during the wound healing process (Fig. 2). At 2 dpi the wound site presented a V-shaped structure with a mean distance across the dense connective tissue most proximally to the coelom of 386 μm; the inside edges of this wound had been layered with scarring tissue. The longitudinal muscles and the inner connective tissue of the dermis were exposed and tissue debris and tattoo ink were present within the surrounding area. The vicinity of the wound had been infiltrated by a considerable number of basophilic spherule-containing cells. Interestingly, the staining pattern near the edges had changed, staining darker shades of fuchsia than in non-injured animals. At 6 dpi, the staining near and at the wound edges reached its uppermost intensity coinciding with a substantial decline in the amount of debris at the injury site and its periphery. This stage was also characterized by having an accumulation of provisional matrix in the wound that served as scaffolding, bridging the separation created in the dermis as a result of the injury. This scaffold stained light fuchsia and was infiltrated by basophilic cells; also noticeable, was the presence of small nuclear bodies. In addition, threads of connective tissue appeared to anchor the muscle terminals to the scar. Fusion of the dermal gap at the injury site was completed by 12 dpi, when the dermal substratum beneath the longitudinal muscles was complete. The location where the circular muscle band had been ruptured was occupied by a dense basophilic connective tissue with large number of tightly packed cells. The segment of connective tissue that was in direct contact with the coelomic contents was nearly completely covered by coelomic epithelium that was contiguous with that of adjacent terminals from the circular muscle bands. Nevertheless, dermal tissue architecture was not similar to that of a normal bodywall. It is noteworthy to mention that from 6–12 dpi the laminae of dermal connective tissue in the areas peripheral to the wound appeared separated from one another, suggesting a diminished cohesiveness. Tissue organization was noticeably improved by 20 dpi, when a solid bridge of connective tissue between the muscle stumps had formed and some muscle fibers were present within it; however, there were areas that still consisted mostly of connective tissue. Additionally, dermal tissue architecture improved dramatically by this stage, appearing much more homogeneous in texture and in cellular composition, resembling more closely that of a normal bodywall. Restoration of all tissue components to a practically normal state was attained at 28 dpi; even so, it is important to mention that even at this stage some tattoo ink can still be observed within the bodywall dermis.

#### B. Phalloidin labeling of the longitudinal muscle

**Figure 3:**
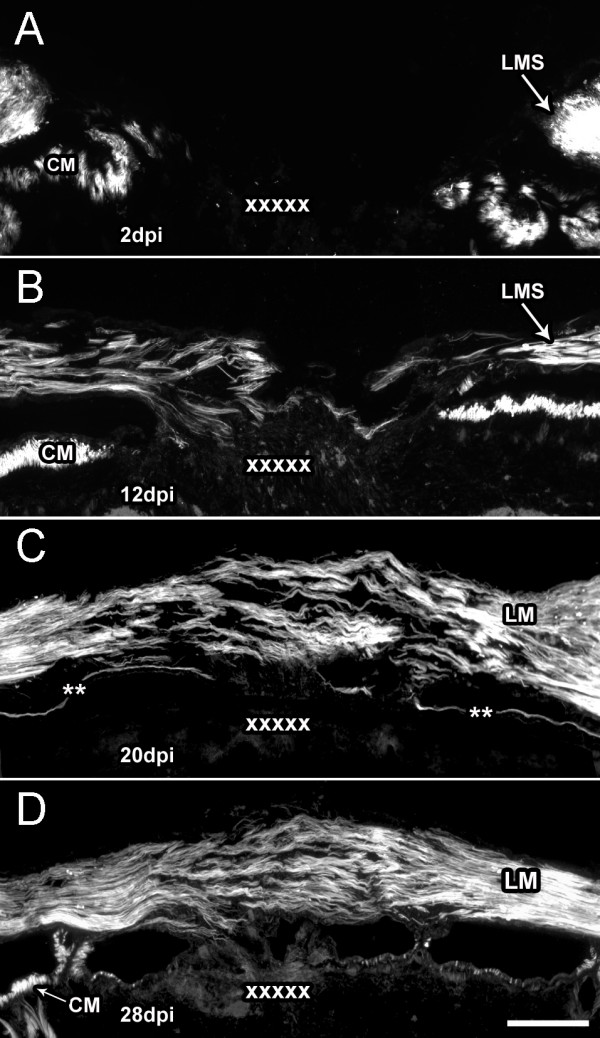
**Wound healing of longitudinal muscle following transection**. Body wall sections from animals at (A) 2, (B) 12, (C) 20 and (D) 28 days post-injury (dpi) were labeled with rhodamine-labeled phalloidin to determine the temporal changes in the longitudinal muscle during wound healing. (A) In the 2 dpi animal, only the muscle stumps created by the wound were labeled. (B) By 12 dpi muscle fibers projecting from the wounded muscle terminals can be found across the injury site. (C) Muscle bundles from opposing stumps can be seen to make contact and cover the injury site by 20 dpi but still show a high degree of disorganization. (D) Muscle organization improves by 28 dpi and the density of the muscle bundles increases; however, organization of muscle bundles is still not fully normal, lacking the compact dense structure found in non-injured muscles (see Fig. 1). CM-circular muscle, LM-longitudinal muscle, LMS-longitudinal muscle stumps. X's mark the injury site; The presence of circular muscle depends on the plane of section since the bands of circular muscle are discontinuous. Asterisks show the area where the circular muscle can be found in nearby sections. Bar = 200 μm.

Although classical histology showed that after a partial musclectomy of the longitudinal muscles, *H. glaberrima *was capable of regenerating muscle fibers across the injury site, the use of Phalloidin allowed for a much more detailed analysis of how the muscle regenerative process occurred (Fig. 3). At 2 dpi the injured longitudinal muscle showed rounded stumps. Discrete muscle fibers could be seen to have extended from some muscle stumps by 6 dpi. However, at this stage they were few in numbers and were only present within 200 um from the stump. Soon after, at 12 dpi, the number and length of fibers had increased and now reached an average of 700 μm into the intervening substratum between opposing muscle stumps. Regenerating muscle fibers seemed to originate from proliferating or migrating myocytes in the muscular tissue, as these fibers were always continuous with the stumps and were never seen isolated as islets of tissue labeled with Phalloidin. A sharp increase in the number of muscle fibers across the injury site was observed by 20 dpi. These were now bundled together and formed a meshwork of muscle bundles between opposing stumps that spanned the wound site; nonetheless, the orientation of these bundles was disarrayed. By 28 dpi, the regenerated segment of muscle possessed tightly packed bundles of fibers that had the same orientation; still, there were few areas within it that were still devoid of muscular tissue, suggesting that some additional time would be necessary for the longitudinal muscle to achieve the compact structure present in normal, non-injured animals.

### Cell proliferation

**Figure 4:**
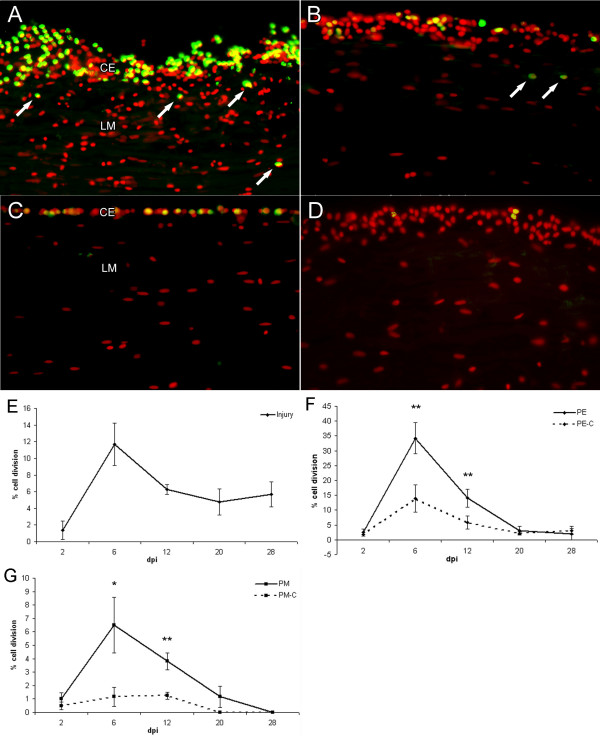
**Patterns of cell proliferation in response to wound healing**. Longitudinal sections were labeled with an antibody against BrdU (green) and Hoescht dye (red) to determine cell proliferation in the longitudinal muscle (LM) and coelomic epithelia (CE). (A) Actively dividing cells were mainly observed in the coelomic epithelia proximal to the injury at 6 dpi. Note that not only the number of dividing cells appears higher but the coelomic epithelium has increased in width. Some cell division is also observed in the muscle layer (arrows) (B) An area distal to the injury site shows much less cell proliferation and a thinner coelomic epithelium. (C) Control (sham-operated) animal at 6 dpi only shows modest cell division and a very thin coelomic epithelium. (D) Minimal cell division is observed at 20 dpi suggesting the stage-dependent role that cell division plays in wound healing. (E) Quantification of cell division at the injury site shows a peak in cell proliferation at 6 dpi. (F) The coelomic epithelia of experimental animals show the largest percentage of diving cells also peaking at 6 dpi. Values of control animals increase slightly but remain significantly lower than experimental animals. (G) Cell division in the longitudinal muscles follows a similar pattern with a sudden peak in cell division at 6 dpi, however the percentage of proliferating cells is much lower than for the coelomic epithelium. CE-coelomic epithelia, LM-longitudinal muscle. PE-Proximal epithelium, PE-C Proximal epithelium-Control, PM Proximal muscle, PM-C Proximal muscle-Control. Bar = 25 μm. Each point represents the mean ± S.E. of at least three animals. *p < .05, **p < .01.

To determine to what extent the process of cell division played a key role in the process of wound healing, dividing cells were labeled with BrdU and measured at different stages (2, 6, 12, 20 and 28 dpi). Dividing cells were easily recognized by double labeling nuclei with Hoescht dye and immunolocalizing the BrdU in cells of animals injected with BrdU the day before. An apparent increase in cell division was observed in the coelomic epithelia and in the longitudinal muscles close to the injury site during the early stages of regeneration when compared to distal tissues or to animals at later stages or controls (Fig. 4). In addition, the number of cells within the coelomic epithelia close to the injury site was observed to increase, probably due to the increase in cell division causing an increase in the width of the epithelia. For purposes of comparison, cell proliferation was quantified at the injury site (mainly connective tissue) and in areas that were both proximal (500 μm) and distal (5 mm) to the injury site (Fig. 4).

#### Injury site

At the injury site, none or little cell division was observed in 2 dpi animals. Cell division began at 6 dpi when about 12% of the cells were observed undergoing division (Fig. 4E). Soon after, by 12 dpi, division levels declined to about 6% and remained constant throughout the subsequent stages.

#### Coelomic epithelia

In the coelomic epithelia, cell division was scarce at 2 dpi; whether one looked at the coelomic epithelium proximal or distal to the injury site only an average of 2 % of cells were observed to be dividing (Fig. 4F). However, at 6 dpi an abrupt increase in cell division occurred both proximally and distally to the injury site. Although both proximal and distal coelomic epithelia showed an increase in cell division, the magnitude in the proximal epithelium was much higher. This increase was even more dramatic if one takes into account that the width of the coelomic epithelium increases during wound healing. In the sham-operated animals there was an increase in cell division in the coelomic epithelia in comparison to normal non-injured animals, but this was still significantly lower than in experimental animals. Furthermore, these control animals did not present the enlarged coelomic epithelium observed in injured animals. A substantial reduction in the extent of cell division took place in both proximal and distal epithelia at 12 dpi, where they exhibited a similar level of cell division. Still, cellular division in the proximal epithelia of injured animals was significantly higher in comparison to control animals. By 20 dpi and forward cell division in the proximal and distal epithelia returned to levels comparable to those of 2 dpi and control animals.

#### Longitudinal Muscles

Little or no cell division occurred within the longitudinal muscles at 2 dpi (Fig. 4G). This was followed by a sudden peak in cell division proximal to the injury site at 6 dpi. Control animals presented a small number of scattered BrdU-positive cells within the proximal longitudinal muscle. These numbers were higher than those seen in animals that had not been sham-operated but still remained significantly different when compared those in experimental animals. At 12 dpi, the difference in cell division in the proximal muscle had diminished but remained significantly higher than in control animals. From this stage forth, cell division continued decreasing steadily until 28 dpi, when no cell division was observable in the proximal muscle. Cell division distal to the injury site remained similar from 2 dpi until 20 dpi; beyond this stage practically no cell division was detected distally.

### ECM remodeling

**Figure 5:**
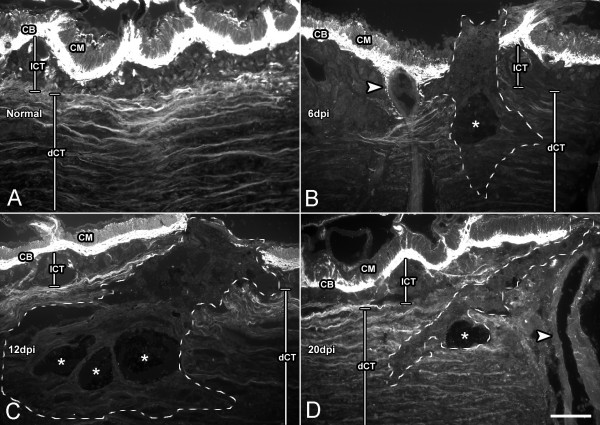
**Changes in collagen immunoreactivity during wound healing**. Longitudinal bodywall sections of (A) non-injured animals and animals at (B) 6, (C) 12, and (D) 20 days post-injury (dpi) labeled with an antibody against holothurian collagen. The normal distribution of collagen found in non-injured animals (A) is disrupted during wound healing. (B) Initially (6 dpi), collagen is lost close to the wound site; dotted line delimits the area depleted of collagen labeling. (C) However, in subsequent stages (12 dpi) the area where collagen disappears extends over a much wider area. (D) Latter stages (20 dpi) show collagen reappearing as the wound heals. All sections were photographed using the same exposure time (30 sec) to depict the actual differences in labeling intensity. CB-collagen band, CM-circular muscle, lCT-loose connective tissue, dCT-dense connective tissue. Arrowheads signal the ampulla within the bodywall; asterisks mark areas where the tattoo ink denotes the injury site. Bar = 200 μm.

Our group has shown that regeneration of the intestinal system is accompanied by a dramatic process of extracellular matrix remodeling. One of the key signals that ECM remodeling is occurring is the disappearance of matrix components, particularly collagen fibers. To determine if ECM remodeling was taking place during bodywall healing we used the monoclonal antibody HgCol, specific for holothurian collagen [16]. In the dermis of a normal bodywall HgCol recognized a lattice of collagen fibers in the loose connective tissue, immediately below the circular muscle band (Fig. 5). In the dense connective tissue of the bodywall, collagen bundles extended through the dermis, parallel to the longitudinal muscles with collagen bundles appearing to be of a much lesser thickness. It is important to note that the collagen bundles recognized by HgCol did not show an even distribution in the dermis; they were closely packed in the area proximal to the coelom than on the area closer to the epidermis. Aside from this, HgCol also recognized the collagenous component of the thin basal lamina of the coelomic epithelia.

#### Bodywall

During bodywall regeneration striking changes in the collagenous content recognized by HgCol were observed in the connective tissue of the dermis. Alterations in collagen distribution were observed as soon as 2 dpi, where most of the collagen bundles near the injury site had disappeared and the remaining few appeared largely disorganized. At this stage, remodeling of the ECM was limited to about a 1 mm radius of connective tissue around the injury site. Collagen disappearance was even more evident at 6 dpi; less collagen labeling was found and the connective tissue area from where the collagen had disappeared had extended as far as 2 mm from the injury site. By 12 dpi reappearance of collagen bundles in the bodywall was seen in the dense connective tissue of the dermis, proximal to the coelomic cavity. Still, the arrangement of collagen bundles was disordered. Reconditioning of the ECM to a state resembling non-injured dermis was observed by 20 dpi; at this stage collagen bundles were again parallel and well-stretched. In spite of this, it is important to mention that, even at 28 dpi, collagen labeling was not present in the connective tissue deposited directly at the wound site.

### Spherule-containing cells

**Figure 6:**
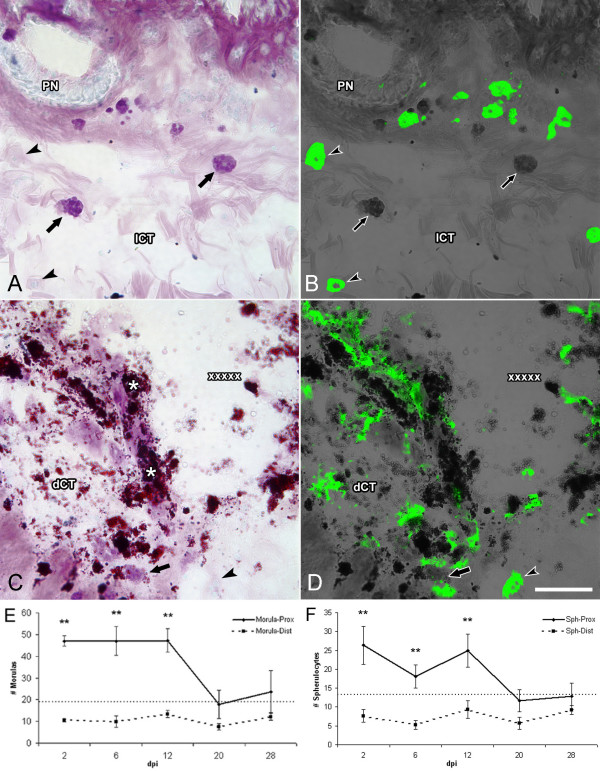
**Changes in spherule-containing cells during wound healing**. Tissue sections (6 dpi) stained with (A) Toluidine blue and (B) Monoclonal antibody Sph2, show two sub-populations of spherule-containing cells: (A) morulas and (B) spherulocytes within the body wall connective tissue far from the injury site. The typical round or oval morphology with well defined spherules is observed. (C-D) At the injury site both cell types undergo dramatic change in morphology, and their spherules are no longer clearly discernable. Sph2-labeled cells, in particular, appear to be releasing their cellular content into the surrounding extracellular matrix. Cell counts show that both spherulocytes (E) and morulas (F) increase in number near the injury site during the first two weeks of wound healing. Their numbers return to levels found in non-injured animals (dotted line) during the third week. A concomitant decrease in cell numbers distal to the injury site is also observed during the first 2 weeks. Dotted line denotes the number of cells found within the bodywall of non-injured animals and of controls. dCT-dense connective tissue, lCT-loose connective tissue, PN- peripheral nerve. Asterisks show the tattoo ink; used to label the injury site; X's denote the injury site; arrows indicate morulas; arrowheads indicate spherulocytes. Bar = 50 μm. Each point represents the mean ± S.E. of at least three animals. **p < .01.

Previous experiments in our laboratory have shown changes in the spherule-containing cell population during intestinal regeneration. In order to determine if similar changes occur during wound healing we followed two spherule-containing cell populations: one labeled with Toluidine blue (named morulas) and one labeled by our monoclonal antibody Sph2 (named spherulocytes). Double labeling experiments showed that when tissue sections were stained with Toluidine Blue and observed under light microscopy, the cytoplasm and spherules of most spherulocytes appeared colorless, while most of the toluidine-stained morula cells were not labeled by our antibody (Fig. 6). Only about 5% of the cells were labeled by both markers.

We observed changes in both cellular populations during the wound healing process. The spherulocyte population appeared to increase near the injury site. Spherulocytes were also observed to go through a striking alteration in their morphology. Cells that were present distant to the injury site, similar to those found in controls and non-injured animals, were round or oval and possessed well defined spherules within their cytoplasm. When these cells were in close proximity to the injury their spherules were no longer discernable and the labeling appeared less intense and seemed to extend into the extracellular matrix. To quantify the changes in the spherulocyte population, the number of cells were counted near the injury site and compared with cell numbers distant to the site and in control animals (Fig. 6E). By 2 dpi, the spherulocyte population already showed a twofold increase in numbers. The changes in cell number and morphology continued during 6–12 dpi. At latter stages (20–28 dpi) cell numbers returned to levels comparable to those of non-injured animals, however, some cells still showed morphological alterations; their spherules were not clearly discernable. An important observation that needs to be emphasized was that while the number of spherulocytes near the injury site increased during the wound healing period, a 32 % decline in their numbers was detected in areas of the dermis distal to the injury site, suggesting that cells were migrating from non-injured areas to the injury site.

The second spherule-containing cell population, the morulas, was also observed to increase in number in response to injury (Fig. 6F). Clustering of these cells near and at the dermal edge created by the incision began as early as in the 2 dpi stage, when a three-fold increase in their numbers could be observed. This peak in cell number was maintained until 12 dpi, after which cell numbers declined to quantities similar to those in normal animals. These cells also showed a parallel decline in their numbers distally to the injury site at stages where an increase from basal levels was registered proximally. A distinctive characteristic of this cell population was that, although they appeared to migrate to the injury site, only a few showed a change in morphology.

### Formation of spindle-like structures (SLS's)

**Figure 7:**
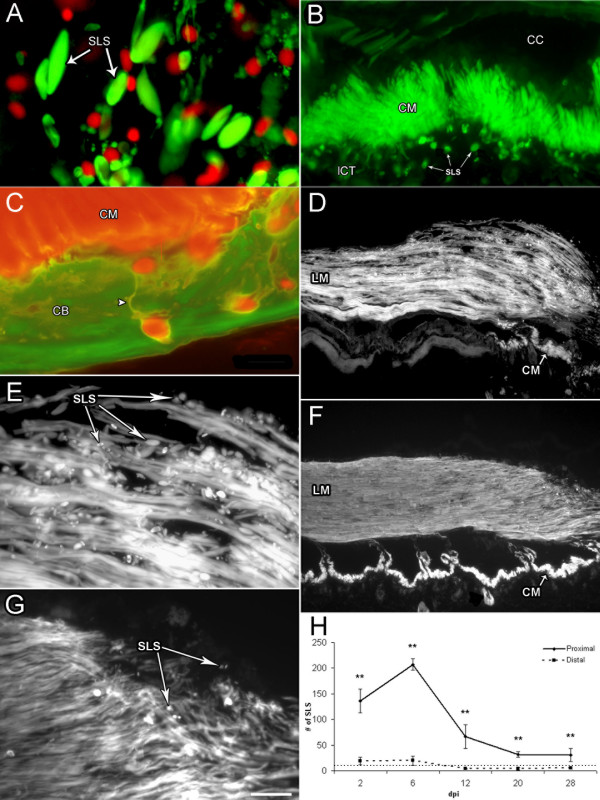
**Formation of spindle-like structures (SLS) by muscle cells during wound healing**. (A) Double labeling of muscle tissue with rhodamine-labeled phalloidin (green) and Hoescht (red) shows the presence of SLS at the injury site. The lack of nuclei within or near the SLS shows the non-cellular nature of these structures. (B) Rhodamine-labeled phalloidin shows the circular muscle band at 6 dpi proximal to the injury site and the released SLS's (arrows) in the bodywall dermis. (C) Double labeling with rhodamine-labeled phalloidin (red) and an antibody against holothurian collagen (green) shows a magnification of the circular muscle and the SLS in the dermal connective tissue of the body wall. Cell extensions or linkers labeled with phalloidin can be observed (arrowhead). (D-G) Longitudinal muscle sections labeled with Rhodamine-labeled phalloidin. (D) At 6 dpi, proximal to the injury site, showing the loose arrangement of the muscle bundles and the presence of SLS's. (E) Magnification of D shows the large number of SLS's present around and within the muscle fibers at this stage. (F) At 28 dpi the muscle bundles are largely organized, and few SLS are observed. (G) Magnification of F showing the reduced number of SLS at the muscle terminals. (H) Quantification of SLS's shows an increase during the first week of regeneration, from 2–6 dpi. At later stages the number of SLSs decreases, although their numbers remain higher than in sham-operated controls or non-injured animals. Dotted line denotes the number of cells found within the bodywall of non-injured animals and of controls. CB-collagen band, CC-coelomic cavity, CM = circular muscle, lCT loose connective tissue, LM-longitudinal muscle, SLS's-spindle-like structure. Arrowhead indicates the SLS phalloidin-positive linker. Bar = (A&C) 10 um, (B) 25 μm, (D& F) 200 μm, (E & G) 50 μm. Each point represents the mean ± S.E. of at least three animals. **p < 0.01.

Previous studies from our laboratory have shown that dedifferentiation of cells within the intestinal mesentery occurs during regeneration of the digestive tract in *H. glaberrima *[19]. One of the hallmarks of this dedifferentiation is the formation and elimination of spindle-like structures (SLS's) made from the contractile apparatus of the dedifferentiating muscle cells. To determine if formation of SLS was also occurring after bodywall lesioning we used the fungal toxin Phalloidin, which binds the polymeric form of actin in the muscle contractile apparatus. Labeling of SLS's using this marker showed the presence of an astonishing amount of SLS's throughout the wound healing process, not only at the injured site, but also in areas distal to the injury. At the injury site, SLS's were present from stage 2 dpi, probably originating from the muscle fibers that had been damaged by the incision (Fig. 7A). As expected, SLS's were shown not to contain nuclei (lack of co-labeling with Hoescht) and were mainly found within the clot or temporary matrix that had formed at the injury site.

#### Circular muscle

Formation of SLS's, was observed within the cells of the circular muscle bands during the healing process. These muscles encircle the coelomic cavity and are surrounded on the dermal side by a dense lattice of collagen fibrils and on the coelomic side by the coelomic epithelium. In the area where the circular muscle band had been ruptured by the injury, SLS's were first noticeable in both muscle sides at 2 dpi. At 6 dpi, they began appearing farther away from the lesion point, on the muscle band contiguous with the terminals created by the injury (Fig. 7B). Peak occurrence of SLS's in the circular muscle bands also took place at 6 dpi, followed by a sudden decline in their frequency at 12 dpi. From this point forward they gradually disappeared to the point of no being detected at 28 dpi. It is noticeable that SLS's ejected from muscle cells into the collagen rich lacunae of the bodywall appeared to remain attached to the dedifferentiating muscle cell by the presence of a cellular extension or linker that labels with Phalloidin and was mainly observed during the 6–12 dpi period (Fig. 7C). In contrast, SLS's discarded to the coelomic surface appeared to be unattached to the remaining muscle cells.

#### Longitudinal muscle

A similar tendency in SLS's formation was observed within the cells of the longitudinal muscle. At 2 dpi, formation of SLS's was restricted to the terminals of muscle tissue adjacent to the lesion. However, at 6 dpi SLS's were present farther away from the lesion area and in the distal regions of the muscle band (Fig. 7D–E). An abrupt deterioration in the muscle organization was also observed, specifically a reduction in the cohesiveness of muscles bundles to the point where individual bundles could be seen. SLS formation decreased at later wound healing stages (Fig. 7F–G).

Quantification of SLS formation was performed to determine the temporal aspects of changes in the muscle structure (Fig. 7H). SLS's were counted per field of view at different wound healing stages. The peak in SLS formation was shown to occur at 6 dpi. Not only were there more SLS's counted per area, but these could be detected as far as 6 mm from the lesion point. By 12 dpi muscle fibers organization was greatly improved and the presence of SLS's confined once more to muscle terminals. From 12 dpi and forth SLS's continued declining, where at 28 dpi little or none could be seen, and muscle organization was virtually comparable to that of an unlesioned animal. If SLS's were present at this stage these were mostly restricted to the injured muscle or to where the overlapping muscle bundles from opposing stumps were in contact. At all stages, formation of SLS's in the longitudinal muscle was predominantly restricted to the coelomic surface of the muscle.

#### Retractor muscles in Papillate Podia (non-locomotory protrusions)

Retractor muscles in the papillate podia, or non-locomotory protrusions, of *H. glaberrima *were also observed to form SLS's in their muscle cells in response to the injury. This phenomenon was more conspicuous in the retractor muscles immediately below the injury. At 2 dpi, these muscles presented an intermediate level of SLS's occurrence. It was at 6 dpi when most SLS's were observed and a large of number accumulated inside the papillate pits. At 12 dpi SLS's had decreased drastically; and from there on little if any SLS's were observed in these tissues.

## Discussion

Here we have described the process of wound healing in the echinoderm *H. glaberrima *and some of the cellular events that take place during this process. We will first discuss the healing process itself in view of what has been described in the literature for other echinoderm species. However, our main interest in undergoing this study was to compare the cellular events that occur during wound healing to those that occur during organ regeneration. By far what has attracted the attention of scientists to the echinoderms has been their capacity to regenerate following autotomy, where the detachment of the limbs or organs serves a defensive function [20]. In most cases regeneration following autotomy involves not only the restitution of particular tissues but the complete formation of an organ or arm. Nonetheless, certain aspects of regeneration, particularly those that occur early in the process and are involved with the healing of the cut or severed surface, can serve as points of comparison to our results.

Therefore, the main part of this discussion will focus on the four events studied: (1) cell proliferation (2) increase in spherule-containing cells (3) ECM remodeling and (4) SLSs formation (and its association with muscle dedifferentiation) and the comparison to intestinal regeneration.

### Wound healing in the holothuria

There are surprisingly few studies describing wound healing in echinoderms and even those that study wound healing show some differences between them. Two studies have focused on cutaneous wound healing: one in *Stichopus badionotus *[21] and a second one in *Thyone briareus *[22]. Both groups of investigators show that wounds heal rapidly (2–3 weeks) with little or no cell division during the early healing stages, but with abundant cell migration. However, they report different findings on the type of cells that migrate and might be involved in the healing process. Our results are consistent in also showing rapid wound healing, little cell proliferation during early stages and changes in cell number that suggest cellular migration toward the wound site. However, our results are not wholly comparable since the previous studies were done following cutaneous injuries that did not affect the musculature while our injury was done on the coelomic side of the bodywall and caused damage to muscular, nervous and dermal tissues. In addition our results show other cellular events that went unnoticed to the previous investigators probably due to, differences in species, in wound type or most probably because of technical limitations.

### Cellular events

#### Spherulocytes/morulas

As mentioned above, there has been some controversy as to the role of the spherule-containing cells in wound healing in holothurians. For the sake of this discussion, we would refer to two types of spherule-containing cells: morula cells, that are labeled with classical histological dyes [21-23] and spherulocytes that are labeled by our monoclonal antibodies [24]. An initial study on the morula cells during superficial cutaneous wound healing in the sea cucumber *Stichopus badionotus *found no significant change in their numbers, thus, suggesting that this cell type does not play an important role [21]. However, more recent works in two other sea cucumber species, *Thyone briareus *and *Eupentacta quinquesemita *question this conclusion [22,23]. They found that numerous morula cells accumulate in the hypodermis of the wound area during the first two days following excision of a portion of the integument [22]. They also report that in incision wounds, the morula cells invade the wound area in large numbers. Similarly, studies of the changes that occur in *E. quinquesemita *during introvert dermis evisceration and regeneration, suggest that morula cells accumulate in regenerating extracellular matrix and provide some of the ground substance material for tissue repair [23].

Our results are in accordance with an important role for morula cells in the wound healing process of holothurians. The increase in number at the injury site during the early stages of wound healing surely suggests that they are somehow involved in the process. Moreover, the fact that their numbers in nearby (non-injured) areas diminish also suggest that morula cells are being actively recruited into the injured area. Differences of these studies with others [21] could be due to the latter only doing superficial injuries or to species differences.

When wound healing is compared to intestinal regeneration one observes, that in contrast to their role in wound healing, morula cells do not appear to play an important role in intestinal regeneration. Although they are present within the regenerating intestine, their numbers actually decrease during the initial stages of regeneration [14].

Spherulocytes appear to be also involved in the process of wound healing. Moreover the changes observed in these cell types during wound healing parallel those observed during intestinal regeneration [24]. In both wound healing and regeneration the number of these cells, localized in the connective tissue, increases. In addition, during both processes they also undergo a dramatic change in their morphology. Complementary to this change in morphology is the alteration seen in the labeling pattern, which is no longer restricted to the interior of the cells but seems to be on the extracellular matrix suggesting that they are releasing their vesicular components into the extracellular matrix. Finally, during wound healing spherulocytes appear to migrate from the adjacent dermis into the wound site. Similarly, during intestinal regeneration, spherulocytes appear to migrate into the regenerating intestine via the mesentery connective tissue [17].

Finally, our results provide for some interesting comparisons between the morula and spherulocyte populations. It has been proposed that the different spherule-containing cell populations reflected cells at different stages of maturation, where the deeply stained cells were younger, and as they matured, their granules were only weakly stained [23]. Based on this, we had previously proposed that spherulocytes were morula cells at a later stage in their maturation [24]. However, the present data questions this interpretation since they show that both morula and spherulocyte populations increase concomitantly, and both appear to migrate into the wound area. Moreover, less than 5% of the cells are labeled with both the antibody and the histological stain. Thus, the data is more consonant with both cell types being independent cell types rather than being the same cell type at different maturation stages.

#### Cell proliferation

The consensus among investigators that have studied holothurians is that little cell proliferation occurs during the early stages of wound healing and that most of the repair in the early stages is accomplished by displacement of cells rather than by proliferation. This has been reported in the sea cucumbers *Stichopus badionotus *and *Thyone briareus *where no significant mitotic figures were observed during the early stages of cutaneous wound healing [21,22]. Our result showing that less than 2% of cells are dividing at 2 dpi certainly fits within what has been found in other studies.

Although the small amount of cellular proliferation that takes place during the early stages of wound healing might seem surprising, this is somewhat similar to what has been found to occur during regenerative events in holothurians. In our previous studies using BrdU labeling during intestinal regeneration in *H. glaberrima*, a small level of proliferation was documented during the early days of intestinal regeneration, while the increase in cellular proliferation occurs between 7–14 days [14]. Similar results have been documented in other species using other techniques; in *Stichopus mollis *by counting mitotic figures [25] and in *Eupenctata fraudatrix *using radioactive thymidine [26-29]. Thus, once again in the sea cucumber, the wound healing events are similar to those found during organ regeneration.

Another resemblance between wound healing and intestinal regeneration, is that in both processes the coelomic epithelium stands out as a center of cell proliferation. This was observed in our previous studies of intestinal regeneration in *H. glaberrima *where the division of the coelomic epithelial cells was much higher than those within the connective tissue [14]. Similar percentages in cellular division of the coelomic epithelia were found in this study following wound healing. The important role of the coelomic epithelia in cell proliferation during regenerative events seems to be a common theme to many echinoderm species, having been shown to occur in crinoid arm regeneration [30] and in arm and pyloric caeca regeneration in starfish [31,32]. Moreover, the proliferation of the coelomic epithelia appears to play a significant role in the restitution of echinoderm tissues since it has been proposed these cells give rise to other cellular types and not only coelomic cells [18,29,33,34].

Cell proliferation also plays an important role in the wound healing of other animals. In mammals, where it has been best studied, a proliferative phase takes place usually 3–5 days following injury [35,36]. Some of the proliferating cells are fibroblast that migrate and proliferate in response to growth factors and eventually play a role in the synthesis and deposition of ECM.

#### ECM-remodeling

There is little discussion of changes within the ECM in previous reports of holothurian wound healing or for that matter in reports of regeneration following autotomy. There are reports of loosening of the ECM fibrils near the wound site [22] and of a zone of degeneration in the deeper collagen bundles [21], but little additional descriptions are provided. Collagen deposition appears to occur rather fast, within a few days of cutaneous wounding in *S. badionotus *[21]. The difference between this report and our findings where, collagen deposition occurs much later might be due to the limited amount of damage in previous studies, where only the epidermis needs to be restored prior to the new ECM being deposited. In contrast, in our studies where other tissues, such as muscle and nerve, need to be healed it might be necessary to maintain a temporary matrix for a longer period prior to the deposition of the permanent one. However, the differences between the previous report and our data might also be explained by technical limitations in identifying particular ECM molecules or changes in ECM, at the time those experiments were performed.

Previous experiments from our laboratory have shown that during regeneration of the intestine in *H. glaberrima *there is a striking remodeling of the extracellular matrix within the mesentery [16]. Among the features of this ECM remodeling are changes in ECM components, activation of matrix metalloproteases and the increased presence of cells involved in matrix degradation and matrix deposition [16,19,24]. One of the hallmarks of the ECM remodeling is the disappearance of ECM collagen, particularly from the mesenterial ECM close to the regenerating intestine. Our results with wound healing show noteworthy similarities to the events that occur during intestinal regeneration. In particular, the disappearance of collagen immunoreactivity in the wounded area and in areas adjacent to the wound, and the temporal scheme in which this occurs. Collagen immunoreactivity diminishes greatly within the two weeks that follow the injury and this decrease occurs not only in the tissue that has been wounded but extends to nearby tissue. During regeneration, collagen also disappears from the mesentery adjacent to the regenerating intestine and a gradient in its disappearance is also observed as one moves from the mesentery edge towards the bodywall.

That ECM changes occur during wound healing of the sea cucumber bodywall is not surprising, since such changes have been associated with the healing of wound in all, if not most, animals where it has been studied. For example, during skin wound healing in mammals, the ECM collagen is degraded and replaced with a temporary matrix [35,37]. This temporary matrix is eventually degraded once the tissues are regenerated and collagen is once again restituted [38]. Similar events have been documented in other organisms following injury [39]. However, one of the consistent findings that differentiate the holothurians from other species is the timing of the response. Collagen disappearance occurs during a 1–2 week period following either injury or evisceration and the reposition of collagen occurs 1–2 weeks afterwards. In contrast in mammals and other species, collagen is degraded and restituted much faster.

#### Cellular dedifferentiation

Recent findings from our laboratory have shown that muscle cells in the mesentery of *H. glaberrima *undergo a process of dedifferentiation during intestinal regeneration [19]. This process can be followed by the appearance of spindle-like structures (SLS's) that are formed from the muscle contractile apparatus. In fact, the formation of SLS's has been shown to be a definite signal of muscle dedifferentiation in echinoderms [27-29,34]. During intestinal regeneration in *H. glaberrima*, these SLS's are formed by the muscle cells located in the mesentery and are then eliminated either into the coelomic cavity or phagocytized by amebocytes in the connective tissue.

There are various similarities between the process of muscle dedifferentiation in intestinal regeneration and wound healing. First, cellular dedifferentiation as determined by SLS's formation occurs during the first and second week following the traumatic event. Second, some of the SLS's are eliminated into the coelomic cavity. An additional similarity is that the dedifferentiation process appears to occur in a gradient, showing high levels close to the injury site and lower levels as one moves distant from the injury site. Such a gradient suggests the presence of a soluble diffusing molecule that might be responsible for muscle dedifferentiation. In this respect, it is interesting that a soluble factor from serum that appears to play a similar role in the dedifferentiation of amphibian myocytes has been partially characterized [40].

Muscle dedifferentiation and formation of SLS's is not restricted to holothurian species. It has been documented in various other echinoderm species undergoing regenerative processes including visceral and longitudinal muscles of sea cucumbers [27,28,34,41,42] and crinoid arm muscle [34,42]. Likewise, elimination of the contractile apparatus and formation of de-differentiated cells from muscle cells has been documented in many other invertebrate and vertebrate species during regenerative procedures. Examples of these are the muscle cell dedifferentiation that occurs during annelid regeneration [43,44] and in amphibian tail and limb regeneration [45-47]. Thus, available data strongly suggest that muscle dedifferentiation is an important component of regeneration processes.

**Figure 8:**
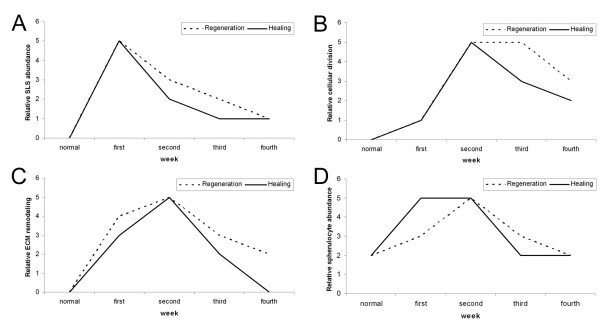
**Comparison of the cellular events that occur during wound healing and organ regeneration**. The four cellular events highlighted in this study were compared to those that occur during intestine regeneration [14, 16, 19, 24]. For this the magnitude of the events was assigned a relative value between 0 and 5 at the various time points where they had been studied. Each event shows a particular expression profile, nonetheless, a stunning similarity is observed between regeneration and wound healing in both the temporal pattern of the event and the peak of occurrence. Additional details provided in the text. (A) Spindle-like structure (SLS) formation, (B) cell proliferation, (C) Extracellular matrix (ECM) remodeling and (D) increase in spherulocyte population.

To summarize the comparison between the cellular events that occur during wound healing and those that occur during regeneration, we have prepared graphs showing the relative extent of the event during four weeks following the injury or evisceration process (Fig. 8). The similarities in terms of magnitude and timing of these events are striking. Except for the increase in spherulocytes all other events follow almost identical temporal sequences in regeneration and in wound healing. The difference in the spherulocytes can be ascribed to the fact that these cells appear to be migrating from the bodywall during the regenerative process and thus, take longer to reach the intestinal primordia. In contrast, these cells are quite numerous within the bodywall and therefore are readily available near the wound site. Nonetheless, it is important to emphasize that spherulocytes are still numerous during the second week of wound injury, thus, although the increase in number begins earlier than in regeneration, they still are present in high numbers at the same time their number peak during regeneration, suggesting a similar role in both processes.

The graphs also serve to highlight the temporal sequence of the evisceration/wound healing related events. It seems, that the cellular event that occurs earlier in both processes is the dedifferentiation of muscle cells. The highest numbers of SLS's are usually observed within the first two weeks and decay thereafter. Other events peak during the second week of regeneration/wound healing. Of particular interest is the fact that little cell division occurs during the days following injury or evisceration and that most of the cell division occurs at the end of the first week or during the second week, thus suggesting that the initial cells involved in wound healing and regeneration processes are mainly cells already found within the tissues or cells that result from the dedifferentiation event. Experiments are in progress to determine the role that the dedifferentiated cell population plays in the regeneration and wound healing processes.

### Comparisons between non-regenerating animals and holothurians

In an essay entitled "What controls organ regeneration?", Davenport [48] states that in order to understand the mysteries of regeneration it will be necessary to determine what separates the human (or mammal) wound healing process from that of animals that are able to regenerate. Our data provides some light into this issue. Two of the cellular events, cell proliferation and ECM remodeling, are observed in non-regenerating species during wound healing. However, there might still be some differences between these processes and those found in animals capable of organ regeneration. For example, cell division usually is documented to occur much faster in mammalian wound healing than what we have documented for holothurians. But in other regenerating species cell proliferation is known to occur widely. In fact, regeneration processes have been traditionally divided into epimorphic processes where high cell proliferation occurs within a blastema and morphallactic processes where little or no cell division occurs and the new organ is formed from pre-existing cells [3,13]. Thus, it is unlikely that the pattern of cell division can serve to differentiate animals capable of extensive regeneration from those whose regenerative properties are limited.

In contrast, there appears to be some notable differences among regenerating and non-regenerating animals in terms of ECM remodeling. In *H. glaberrima *ECM remodeling extends over weeks rather than days and the peak in both collagen disappearance as well as in its deposition appears to be much later than during wound healing in mammals. ECM remodeling has been highlighted as one of the mechanisms that is found to occur widely in animals capable of organ and limb regeneration. It has been shown to occur during newt limb regeneration [39,49-52] rat liver regeneration [53], Hydra head regeneration [54,55] and zebrafish fin regeneration [56]. In mice, ECM remodeling, and in particular the activity of the matrix metalloproteinases, has been shown to correlate with the regeneration ability of two mice species: those where the activity of the enzymes is higher and last longer show better healing of ear wounds [57]. Therefore, the timing and extent of ECM remodeling might be an important factor associated with the enhanced regeneration capacities.

Comparisons between regenerating and non-regenerating animals, in terms of the population of spherulocytes are not practical at this time. First, because, their role in holothurians is not clear. They have been associated to the production of a transitory ECM [23,24] but have also been implicated in defense mechanisms [58-60]. Second, because it is uncertain if they have analogous cell type in other species. Some investigators have suggested they are similar to mast cells [21,22,24] but this possibility remains undetermined. Nonetheless, the association of this cell type with the process of wound healing and regeneration in holothurians is worth further exploration, particularly in view of their capacity to release their vesicular contents which might include not only ECM components but growth factors, cytokines and other molecules associated with the cellular events that have been observed.

Out of the four cellular events that occur during holothurian wound healing and regeneration, the only one that appears to be prominent in regenerating species but not to occur in poor regenerators is the dedifferentiation of muscle cells. As discussed above, this process has been documented in various animal species ranging from annelids to amphibians. Moreover, the process could be the source of stem cells that eventually provide the different cell phenotypes necessary for the formation of the new organ [61,62]. Such a possibility has been explored by the laboratory of Tanaka [6,47] showing that, in amphibian tadpoles, cells that originate from muscle cell dedifferentiation gave rise to a variety of cell types. The process of dedifferentiation is not limited to muscle cells. In fact, other cells have been shown to dedifferentiate during the process of regeneration. The best documented is the dedifferentiation of the dorsal iris cells to produce the new lens in salamanders [5]. While recently, it has been shown that dedifferentiation of digestive cells into blastema-like cells occurs in Hydra during head regeneration [63]. Therefore, cell dedifferentiation might be one of the key processes that separate good regenerators from poor regenerators. Thus as has been suggested by Han and colleagues [61] "gaining access to developmentally regulated programs (dedifferentiation) lies at the heart of the regeneration problem in mammals".

## Conclusion

The cellular events that occur during wound healing in *H. glaberrima*, namely the pattern of cell division, increase in the spherulocyte population, ECM remodeling, and muscle dedifferentiation are similar to those that take place during intestinal regeneration. Thus, our results show that, at least in *H. glaberrima*, but probably in most echinoderms that undergo organ or arm regeneration following autotomy, the same processes that are used for regeneration of complex structures are used for wound healing. Of the four events, muscle dedifferentiation appears to be a common mechanism to animals that posses a high regeneration capacity thus suggesting that this mechanism is of great importance to the regeneration process in metazoans.

## Methods

### Animals

*Holothuria glaberrima*specimens were collected in the northeast coast of Puerto Rico and maintained in the laboratory in recirculating seawater aquaria with constant oxygenation.

### Surgical procedures

Animals were eviscerated by injecting 0.35 M KCl (3–5 ml) into the coelomic cavity. One hour following evisceration, animals were anesthetized by submersion in 0.2% 1,1,1-Trichloro-2-methyl-2-propanol hydrate (chlorobutanol) in sea water. Once they were fully relaxed, approximately 10 min, exposure of the longitudinal muscle was achieved by pushing the bodywall from the outside with a blunt rod through the cloaca. This left exposed nearly 2 cm of the dorsal wall that faces the coelomic cavity, hence, providing accurate and easy access to the longitudinal muscle. The injury was performed by making a perpendicular incision with a scalpel between the longitudinal muscle bands, 3–4 cm anterior to the cloaca and about 2 cm from the point of stress. Injured tissues included longitudinal muscle, circular muscle, radial nerve, water canal, bodywall dermis and coelomic epithelia. Control animals were subjected to the same inversion procedure except that no lesion was made. In the experimental animals, the injury extended 1–1.5 mm into the inner dense layer of the dermis and the lesion site was then marked with red tattoo ink to be able to identify it later during dissection. Animals were returned to the aquaria and allowed to heal for different days (2, 6, 12, 20, and 28) post-injury (dpi). Before sacrificed, animals were again anesthetized with chlorobutanol, dissected, pinned and initially fixed with 4% paraformaldehyde poured directly into the dissecting dish for 30 min. The area around the incision was then localized and a 6 mm × 12 mm rectangle was dissected and kept overnight in 4% paraformaldehyde. Tissues were maintained in 30% sucrose until use. All dissections were carried out using a Wild-M5A dissecting microscope (Wild Heerbrugg, Switzerland).

### Histology

Bodywall fragments were embedded in OCT embedding medium (Tissue Tek OCT; Miles Inc.), frozen at -20°C and longitudinally sectioned (20 μm) in a cryostat (Leica CM 1900). Sections were recovered on poly-lysine-treated slides, left to dry for at least 1 hr under cold air and stored until use. All sections used for data analysis were from bodywall tissues not farther away than 200 μm from the medial plane of the longitudinal muscle pair, where the distance across the injury was greatest. Proximal regions were 0.5 mm from the injury site while distal regions were 5 mm away, approximately. Images were recorded and analyzed using the MetaVue software (version 6.0; Universal Imaging, Inc.).

### Histological analyses

#### Morphometric analysis – muscle regeneration

To assess the progress of wound healing, the distance between muscle stumps labeled with phalloidin were measured from 10× micrographs at different stages (2, 6, 12, 20, 28 dpi). For each specimen, five to ten sections were analyzed under fluorescence microscopy and the shortest distance, for each animal, between the muscle terminals was recorded.

Also, the extent of muscle regeneration was quantified by measuring the length of the regenerated fiber from the muscle stump to the farthest point in the regenerating front. For each animal, five to six measurements were taken and the mean value was calculated for each animal, pooled with other values for that same stage, then a final mean was calculated.

#### Chemical Dyes – toluidine blue

Slides with longitudinal sections of the bodywall were used to determine the temporal progress of healing in the injured tissues. Cryostat tissue sections were treated with Toludine blue as described by Presnell and Schreibman [64]. In brief, the sections were rinsed in PBS for 2 min, and then immersed in dye solution for 2 min. They were then rinsed in PBS, mounted in buffered glycerol phosphate and viewed under the microscope. In some cases, staining was done on tissue sections previously processed for immunohistochemistry (see below).

### Immunohistochemistry

The immunohistochemical procedures used have been described elsewhere [14-16,18,65] Cryostat tissue sections were treated with goat serum (1:50), permeabilized with 0.5% Triton X-100 and incubated overnight with the corresponding primary antibody: Anti-BrdU (1:5, Amersham), Sph2 supernatant [24] or HgCol supernatant [18] in a humid chamber. The following day, sections were rinsed in PBS and treated with the FITC-labeled goat-anti-mouse secondary antibody (1:50, BioSource Int.). For double labeling, phalloidin-rhodamine (1:200, Sigma) was added together with the primary antibody. Sections were rinsed again in PBS and mounted in buffered glycerol. Double or triple labeling with Hoechst nuclear dye (Sigma) was done by immersing slides in 1 μM Hoescht for 15 min after the primary antibody [14,18]. Sections were examined and photographed using a Nikon E600 microscope equipped with FITC, R/DII and DAPI filters. Images were recorded using the MetaVue software (version 6.0; Universal Imaging, Inc.).

### Spherule-containing cell counts

The number of spherule-cells labeled by immunological (Sph2) and histological (Toluidine blue) stains were quantified by counting the number of labeled cells in an area of 280 mm^2 ^(the microscope field of view using the 40× objective). For these experiments we used longitudinal sections of the bodywall labeled with Sph2 and stained with Toluidine Blue simultaneously; previous testing confirmed that the dye Toluidine Blue did not affect Sph2 labeling. Cell numbers were obtained from the inner (dense) layer of dermis proximal and distal to the injury site at various stages during the wound healing period. Cell counts were done in two fields of view chosen at random for each corresponding area in every animal and in at least three animals per stage of regeneration. Student's t test and ANOVA's were used for statistical analyses.

### Quantification of spindle-like structures (SLS's)

Previous work from our laboratory and that of others has shown that muscle cells can dedifferentiate forming spindle-like structures [19,27]. To examine if muscle cell de-differentiation was taking place during wound healing and the possible extent of this occurrence, the numbers of SLS's present in longitudinal muscles of similar width were tallied. Measurements were made at all stages, both distally and proximally to the injury site, in two fields of view (20×) for each corresponding area in at least three animals per stage. Student's t test and ANOVA's were used for statistical analyses.

### ECM remodeling – collagen degradation

Analysis of the changes in the extracellular matrix during wound healing was possible on account of the HgCol antibody [16]. For this purpose, 10× micrographs of labeled tissue sections of the wounded areas were taken with the microscope under the same illumination conditions and time exposure settings. To quantify collagen disappearance, the total area in the dermis of the bodywall with no immunoreactivity for collagen was determined. Images were recorded and analyzed using the MetaVue software (version 6.0; Universal Imaging, Inc.).

### Proliferation Assays

The temporal and spatial distribution of cell proliferation in the healing animals was assayed using BrdU incorporation. Animals were injected intracoelomically at 2, 6, 12, 20 and 28 dpi with 100 μl of a 1 mg/ml BrdU solution (approximately 0.01 mg/g wet weight) and sacrificed 24 hrs after. Tissues were fixed and treated as described above for immunohistochemistry. Antibodies against BrdU (Amersham) were used to detect cells that had incorporated BrdU. Immunocytochemistry was performed as described above including an additional bath of 0.05 N HCl for 1 h prior to addition of the BrdU antibody, to improve the accessibility of the antibody to the BrdU epitope, followed by an additional 15 min PBS wash.

The labeling index was obtained by counting the number of cells labeled with BrdU divided by the total number of Hoescht-labeled nuclei per microscope field of view. For the longitudinal muscles and their epithelia a mean value for two corresponding areas in each animal was calculated. At least 3 different animals were used for each wound healing stage. Measurements were made at the injury site as well as proximal and distal from the injury site.

## Authors' contributions

JSMR carried out most of the experiments and helped in the analyses of data and preparation of the manuscript. JEGA conceived the study, participated in its design, data analyses and helped in manuscript preparation. All authors read and approved the final manuscript.
